# Photonic and Optoelectronic Devices and Systems, Third Edition

**DOI:** 10.3390/mi16121384

**Published:** 2025-12-06

**Authors:** Muhammad A. Butt

**Affiliations:** Institute of Microelectronics and Optoelectronics, Warsaw University of Technology, Koszykowa 75, 00-662 Warsaw, Poland; ali.butt@pw.edu.pl

The rapid evolution of micro- and nanoscale technologies continues to shape modern scientific development across photonics, semiconductor devices, sensing, displays, and optical systems [1,2,3,4]. This Special Issue of *Micromachines* brings together ten contributions that collectively demonstrate the breadth and vitality of current research in these areas. Among them is one comprehensive review on transverse mode adaptive control based on photonic lanterns, accompanied by nine original research articles that address important advances in optical waveguides, high-purity and three-dimensional detectors, metasurface-based photodetection, electrowetting display optimization, avalanche photodiodes, and compact lens design for augmented reality. This editorial highlights the core contributions of each paper and reflects on the collective impact of the Issue.

The review article by Lu et al. provides a timely and forward-looking exploration of transverse mode adaptive control using photonic lantern technology [contribution 1]. The authors clearly articulate the limitations of existing mode control techniques and present photonic lanterns as a powerful pathway for achieving high-purity and rapidly reconfigurable multimode outputs in fiber systems. Their discussion spans device structures, fabrication approaches, mode evolution mechanisms, and integrating adaptive algorithms. The review underscores that photonic lantern-based adaptive systems offer compactness, fast response, low loss, and excellent scalability. The authors also identify impactful application fields that include suppressing transverse mode instability, mode division multiplexing, particle manipulation, and advanced spectral measurement. This study serves as an essential reference for researchers engaged in high-power fiber laser engineering, photonic integration, and adaptive optical control.

The nine original research articles in this Issue present significant advances across diverse domains. The study by Butt et al. investigates silicon nitride (SiN) ridge waveguides for enhanced refractive index sensing, combining numerical modeling with experimental validation [contribution 2]. Through a precise analysis of the effective refractive index, evanescent field ratio, and propagation losses, the authors demonstrate how waveguide geometry and wavelength selection can be leveraged to achieve improved sensitivity. Their experiments using a racetrack ring resonator reveal a marked increase in sensitivity at longer wavelengths, offering meaningful insights for the design of highly responsive photonic sensors. This study led to the further development of more advanced refractive index-sensing devices [5].

High-purity germanium drift detectors are examined in the study by Wang et al., who introduce an innovative design to address capacitance challenges in conventional detectors [contribution 3]. By using a small-area central cathode surrounded by concentric anode rings linked by a resistive chain, the detector achieves a capacitance determined solely by the collecting electrode rather than the overall device size. TCAD simulations confirm smooth electric potentials, directed lateral drift channels, and strong collection performance under heavy ion irradiation. This research provides a promising new direction for radiation detection instrumentation used in dark matter searches, high-energy physics, and space-based sensing.

Yoo et al. present a significant improvement in inverted organic light-emitting devices using lithium-doped magnesium zinc oxide nanoparticles as the electron injection layer [contribution 4]. The nanoparticles exhibit reduced particle size, modified energy band positions, and enhanced surface and electrical characteristics as lithium content increases. OLED devices fabricated with these modified layers achieve markedly higher external quantum efficiency through improved charge balance. Notably, the maximum efficiency reaches more than 20%, demonstrating the effectiveness of lithium doping as a strategy for tuning electron injection properties.

Three-dimensional (3D) silicon detector technology is the focus of two contributions. The first, by Zhu et al., proposes a polysilicon fill-strengthened etch through a 3D trench electrode detector that resolves the issue of non-uniform fields and dead zones in traditional trench designs [contribution 5]. Using double-sided etching and filling, the authors achieve a fully penetrating trench structure that improves electric field symmetry and reduces depletion voltage. Their simulations show extremely low leakage current, low capacitance, and efficient charge collection, indicating strong potential for demanding applications in nuclear radiation detection and particle physics.

The second detector-related paper by Liu et al. introduces a novel through-type semi-spherical electrode detector built on an SOI substrate [contribution 6]. The unique geometry equalizes horizontal and vertical dimensions to create a quasi-spherical electric field distribution. The result is a device with remarkably small capacitance, low depletion voltage, and excellent isolation between units, which is especially valuable for high-resolution X ray photon counting. This design highlights the valuable synergy between structural creativity and device physics modeling.

Cheng et al. contribute a design for a long-wave infrared fully polarized HgCdTe photodetector that employs a silicon metasurface to enable both circular and linear polarization resolution within a single compact platform [contribution 7]. The study integrates COMSOL (version 5.6) simulations to reveal how chiral silicon nanostructures and linear gratings work together to decode Stokes parameters without multilayer stacking. The demonstrated extinction ratio of thirty decibels for circular polarization indicates impressive performance. This effort expands the possibilities for compact integrated polarization-sensitive detection in spectroscopy, thermal imaging, and quantum information processing.

Complementing these studies, Yang et al. offer an electrowetting display driving strategy designed to reduce luminance instability caused by overdriving pulses [contribution 8]. The proposed three-stage pulse incorporates an overdriving phase for rapid contraction, a linear switching phase to suppress luminance glitches, and a final driving phase. Both simulations and experiments confirm that this method eliminates luminance instability while simultaneously increasing brightness and decreasing response time. This paper presents an important contribution to the advancement of high-quality reflective display technologies.

The article by Cheng et al. presents a three-terminal silicon germanium avalanche photodiode with a breakdown voltage of only 6.8 volts and an impressive gain bandwidth product of more than 1300 gigahertz [contribution 9]. The device employs a separate absorption and multiplication structure and integrates a silicon nitride waveguide for lateral optical coupling. Simulations show excellent responsivity, high-speed modulation capability, and clean eye diagrams at 100 and 200 gigabits-per-second data rates. This study holds strong relevance for silicon photonics-based receivers in advanced communication systems.

Finally, the compact collimating lens system introduced by Sun et al. demonstrates a practical solution for generating a fifty-degree field of view in lightguide-based augmented reality glasses [contribution 10]. The design uses four plastic aspherical lenses to achieve high optical quality, low distortion, and a very small form factor compatible with a 0.32-inch microdisplay. With a total volume below one cubic centimeter, this optical module advances the goal of lightweight and unobtrusive AR eyewear.

Together, these ten papers offer a valuable snapshot of progress across micro- and nanoscale device engineering [6,7]. They reveal a consistent push toward compactness, efficiency, precision, and integrability across materials and platforms. This Special Issue demonstrates the increasing convergence of photonics, semiconductor technology, sensing, and display innovation, and we hope it will inspire continued research that bridges these diverse yet synergistic fields.
